# Predictive Processing in Sport and Exercise Science: A Scoping Review and Theoretical Overview

**DOI:** 10.1186/s40798-026-01092-z

**Published:** 2026-08-17

**Authors:** David J. Harris, Callum A. O’Malley, Damian Beck, Stephan Zahno, Thomas Arthur

**Affiliations:** 1 Department of Public Health and Sport Sciences, Exeter Medical School, University of Exeter, Exeter, UK; 2 Institute of Sport Science, University of Bern, Bern, Switzerland

**Keywords:** Active inference, Bayesian, Skill acquisition, Free energy, Performance

## Abstract

**Background:**

This scoping review explored the application of predictive processing in sport and exercise science, examining how cognitive and computational models have been used to understand perceptual-motor skills, decision-making, and physical performance. The review provides a tutorial introduction to key concepts in the predictive processing literature and maps the extent of research in this area, identifying key domains of application and the level of empirical support.

**Methods:**

A systematic search of EMBASE, Medline, PsycINFO, and SPORTDiscus databases was performed, adhering to JBI methodology and the PRISMA Extension for Scoping Reviews.

**Results:**

Twenty relevant empirical studies were discovered across all domains of sport and exercise science. Despite promising evidence, the review also uncovers significant gaps, particularly in comparing predictive-processing-specific mechanisms to alternative theories and exploring the broader implications of predictive processing for skill acquisition and training interventions.

**Conclusions:**

Based on these findings, the review outlines recommendations for future research, calling for more targeted investigations that empirically test predictive processing predictions and an expansion of this approach to new areas of sport science.

## Key Points

- This scoping review examines how the framework of ‘predictive processing’ has been applied in sport and exercise science.
- While the review identifies promising evidence in favour of the approach, it also highlights gaps, particularly in testing predictive processing against alternative theories and its implications for training.
- Recommendations are provided for future research, emphasising the need for more targeted empirical studies and broader applications in sport science.

## Introduction

Predictive processing is a prominent idea that has gained significant attention within systems neuroscience [1–3]. This far-reaching approach suggests that perception, movement, and cognition involve internal generative models (or representations) and predictive (and prediction error-minimising) neurobiological dynamics [4–7]. This idea builds upon growing evidence that basic features of neural activity and behaviour are well explained by the principle that the brain encodes predictions about sensory inputs and deviations from those predictions. This evidence ranges from encoding of predictions as early as the retina [8] to fundamental features of visual processing [9] and neuronal message passing [1], decision-making [10, 11], interoception [12, 13], effort [14], fatigue [15], pain [16, 17], visual attention [18], clinical psychiatry [19], motor expertise [20], imagery [21], and choking under pressure [22].

Success in these diverse domains suggests predictive processing may be a valuable explanatory framework for many areas of sport science, where interdisciplinary research concepts and applications are crucial. Indeed, predictive processing frameworks are potentially capable of uniting models and data across domains, using common mechanistic principles. While recent studies have initially explored predictive processing in sports and complex sensorimotor tasks [15, 23–25], this work is in its infancy and there have been few attempts at synthesising evidence between different investigatory domains. Another barrier to development stems from an often-limited understanding of the diverse and sometimes conflicting terminology used across predictive processing frameworks, which complicates efforts to design empirical tests and apply these concepts to sport and exercise contexts. Accordingly, the present review aims to offer an accessible but detailed introduction to predictive processing, before evaluating current applications in sports science via a scoping review of the existing literature. We consider what empirical studies have been conducted in sport science, which subdomains they focus on, and what promising directions for future research and development lie ahead.

### Theoretical Overview

The Predictive Processing Framework (PPF) views the brain and central nervous system as a dynamic prediction engine which continuously generates and updates hierarchical models of the environment by comparing incoming sensory inputs with top-down expectations [5, 6, 26, 27]. The notion that the brain acts as some form of prediction machine has a long history in both the psychology and physiology literature [28–30]. According to the specific framing proposed by the PPF, the brain maintains internal ‘generative’ models that specify probability distributions over hidden causes and the sensory consequences they produce. By inverting these generative models, the brain can infer the most likely causes of its sensory input. A critical motivator for this view is that we only ever access the external world through noisy and ambiguous sensory signals [31]. Consequently, perceptual states do not map directly onto sensory signals, nor do sensory signals have a direct one-to-one correspondence with their causes. Rather, from a Bayesian perspective, the brain’s main task is to select the most optimal hypothesis about the world by integrating sensory inputs with context-relevant prior beliefs. The relative influence of priors versus sensory evidence depends on their precision (inverse variance)—that is, when sensory signals are noisy or ambiguous, priors exert stronger influence on perceptual inference. This is nicely illustrated by perceptual illusions (e.g., the McGurk effect or hollow face illusion), which demonstrate how strongly held priors can dominate perception when sensory information is ambiguous.

**Fig. 1:**
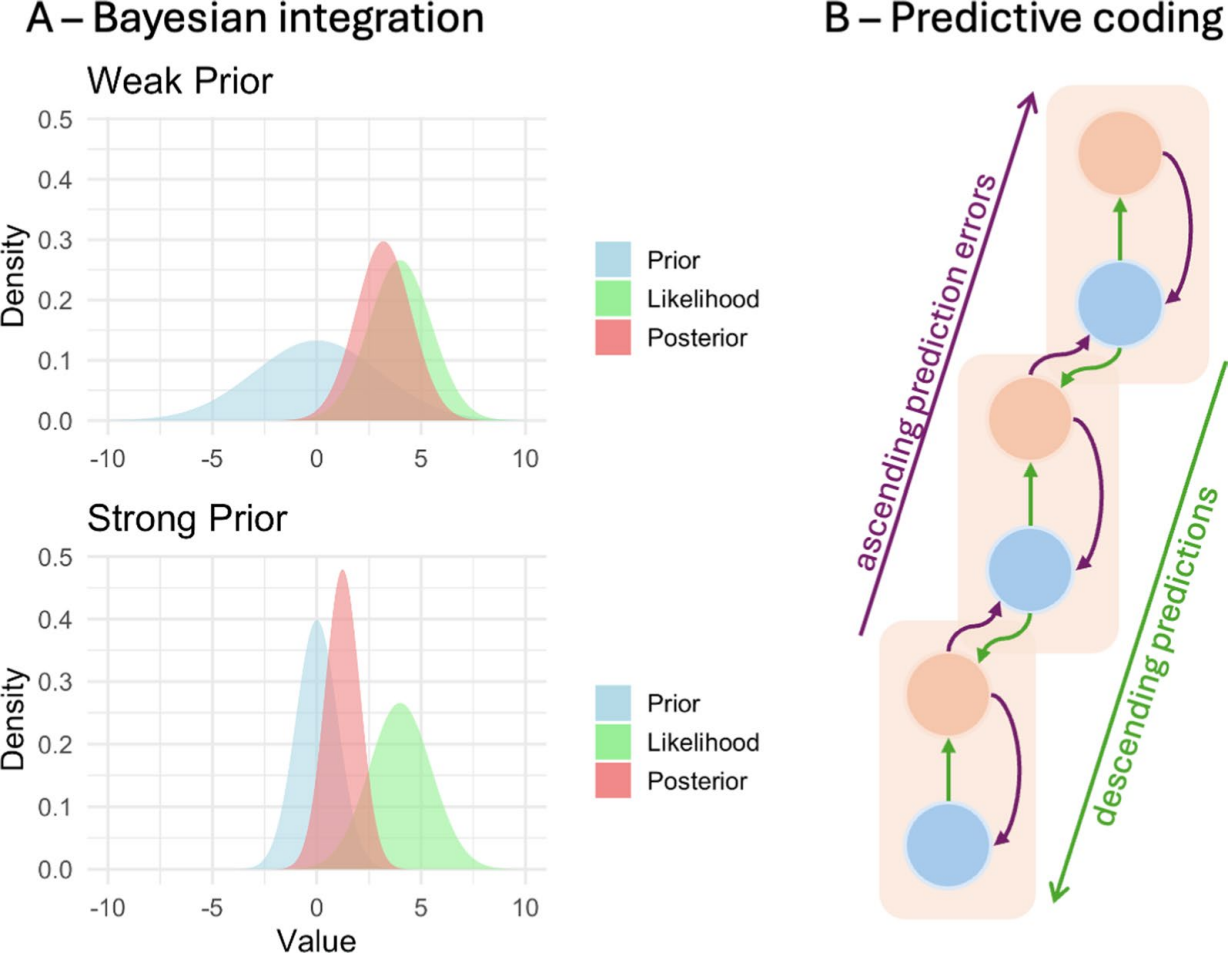
Predictive processing in the brain: **A** Bayesian integration (combining multiple sources of information to obtain a posterior belief of the state of the world); **B** a proposed mechanism of predictive coding through hierarchical neural networks. Panel **A** illustrates the principles of Bayesian integration, showing how different priors influence the resulting posterior distribution over a world state (e.g., opponent action direction, ball trajectory). In the top panel, a weak prior (with a larger variance) is combined with the likelihood, resulting in a posterior that is heavily influenced by the likelihood. In the bottom panel, a stronger prior (with a smaller variance) biases the posterior more toward the prior belief. This demonstrates how the strength of prior information affects the final inference. Code to reproduce this plot is available online: (https://osf.io/mtynv/). In panel **B**, blue ‘state’ units pass prediction errors down the hierarchy and receive error signals from the error units at lower levels, causing them to revise their predictions. Each layer passes prediction error within its own layer (orange to blue) and to the layer above. The goal of the system is to effectively suppress the layer below by removing the error signals being passed back

Crucially, to perceive the world accurately and act effectively, the brain must seek to make suitable predictions about future events, the state of the body it inhabits, and the courses of action that will fulfil its goals [5]. To do so, internal predictive models are constantly revised and refined with newly acquired information. Specifically, prior beliefs are iteratively developed through experience and, in turn, used to generate dynamic predictions that are disseminated via top-down pathways in the brain [32]. These predictions converge with bottom-up sensory input, and any discrepancies between these signals (known as ‘prediction errors’) are used to revise the current hypothesis, resulting in increasingly accurate prediction-making (see Fig. 1 for a visual representation). For instance, it is only after years of practice that a batter in cricket is able to effectively anticipate a rapidly approaching delivery. Here, by predicting the trajectory of the ball through a combination of top-down knowledge (e.g., about a particular delivery type) and bottom-up perceptual information (e.g., relating to the rotation, angle, and speed of the ball), the batter can promptly decide how to play a suitable shot. Critically, though, this batter will not perceive the surrounding world as it truly is; rather, they will be using a best estimate of the external environment that balances observed sensory inputs with their prior probability. Hence, in situations that are unexpected or surprising (e.g., when a bowler effectively disguises a slower ball), they may be inclined to respond based on what is most likely to happen, rather than respond to current sensory cues (e.g., causing the batter to play a shot too early and miss the ball).

The rise of different incarnations of the PPF unfortunately confuses matters. For instance, Andy Clark’s Embodied Predictive Processing [4, 26] emphasises the role of the body and environment in shaping cognitive processes (e.g., embodied, embedded, enacted, and extended cognition), adopting terminology like ‘probabilistic affordances’. Meanwhile, other conceptualisations are more heavily epistemic, inferential, and representational, leaning heavily on computational modelling to describe cognition [33]. Moreover, some conceptual accounts argue that the PPF can be more closely aligned with enactive and ecological accounts of perception and action that may even reject cognitive representations (e.g., [34–36]). Despite these wide variations in conceptual terminology and/or framing, a critical commonality emerges across the field; that is, the universal principle that predictions are crucial for shaping our interactions with the world around us.

Given these conceptual and terminological divergences, the present scoping review adopts a deliberately neutral position with respect to different theoretical commitments within the PPF. We employ terminology broadly shared across the literature—such as ‘prediction’, ‘prediction error’, and ‘hierarchical processing’—while remaining agnostic to more nuanced mechanistic claims.

### Pertinent Concepts

There is substantial ambiguity surrounding terminology and the various interrelated concepts within the PPF, which may pose a barrier to applying these ideas to sport science. To clarify these complexities, we summarise how some different theories and ideas associated with the PPF are interconnected.

#### The Free Energy Principle

The PPF connects closely with the free energy principle [5, 27], which states that biological systems strive to minimise free energy—essentially, prediction error—over time. While the PPF focuses on the mechanisms of model updating and error correction in the brain, the free energy principle provides a broader theoretical framework linking these mechanisms to the overall goal of maintaining homeostasis. For instance, when body temperature rises during exercise, the brain computes the difference between an individual's predicted set-point and their current sensed temperature. This prediction error triggers sweating to cool the body down, simultaneously minimising the error and maintaining homeostasis. In this way, the free energy principle provides a unifying principle that integrates the PPF with a broader biological imperative: to minimise uncertainty and maintain a coherent, adaptive interaction with the world.

#### Predictive Coding

Predictive coding is a specific mechanism or model within the PPF. It describes how the brain enacts predictive processing through hierarchical neural networks [1, 8, 9]. In predictive coding, higher levels of the brain's hierarchy, which process increasingly abstract representations, generate predictions about sensory inputs, and these predictions are compared with actual sensory inputs at lower levels (backward connections). The resulting prediction error is then sent back up the hierarchy (forward connections) to update the predictions, leading to a continuous cycle of prediction and error correction. Consider a tennis player returning a serve. Higher-level brain regions generate predictions about the ball's trajectory based on the opponent’s body position and serving pattern (backward connections). These predictions are compared with actual visual input of the ball’s flight at lower sensory levels. Any mismatch—such as the ball arriving faster than expected—generates a prediction error that propagates upward (forward connections) to update the higher-level predictions, enabling rapid adjustment of the motor response. Prediction errors are weighted based on the confidence (or “precision”) that the brain assigns to the sensory input versus the prediction, allowing it to prioritise certain errors over others. Figure 1B illustrates a simplified version of the proposed mechanism of predictive coding in the brain, as well as a conceptual illustration of the general principles of predictions and prediction errors in a hierarchy.

#### Active Inference

Active inference can be most succinctly described as the PPF, or the free energy principle, applied to action [27, 37]. Active inference proposes that the brain not only minimises prediction errors by revising its beliefs to make more accurate predictions, but also actively engages in behaviours (decisions or physical movements) to sample the environment in ways that fulfil its predictions (i.e. inferring actively). This minimisation of surprise (or free energy) can happen across long or short timescales. Short-term surprise can be minimised by acting to directly influence the state of the world—e.g., a cricket batter moving to intercept the ball before it bounces to remove the uncertainty associated with an unknown level of spin off the pitch. To minimise surprise over the long term, agents may deliberately seek out new information (potentially *increasing* short-term surprise) to improve long-term predictions—e.g., the batter might take calculated risks early, playing unconventional shots or moving around the crease, gathering data on factors like swing or bounce. This balance between short- and long-term minimisation of surprise is sometimes referred to as an ‘explore–exploit trade-off’ [38]. Active inference provides a unified explanation for perception and action and would best be described as a *framework* itself, but could be considered a *theory* in its application to detailed models of motor control [39, 40].

#### The Bayesian Brain

The term ‘the Bayesian Brain’ [41, 42] is also used in related research, often interchangeably with ‘predictive processing’. The Bayesian brain extends the ideas of the PPF by explicitly framing the brain as performing probabilistic inference. According to this perspective, cognitive processes can be understood as a series of probabilistic updates, where the brain continuously refines its internal models according to Bayes' rule. Here, Bayes' rule tells us that our new belief should be proportional to our old belief multiplied by how consistent the evidence is with that belief. The Bayesian Brain theory thus provides a formal, probabilistic account of how predictive processing operates. Key testable hypotheses arising from this theory include that neural activity should reflect prior-likelihood integration weighted by uncertainty, prediction errors should propagate through hierarchical networks, and perception should be systematically biased toward priors under ambiguous conditions.

#### Bayesian Integration

Bayesian integration refers to the process of statistically optimally combining multiple sources of information to estimate an uncertain state of the world [43, 44]. For example, when playing tennis, vision alone can only provide a noisy, uncertain estimate of the ball’s location. Bayesian integration suggests that humans combine incoming sensory input (likelihood) with prior expectations (prior), weighting each source according to its reliability to derive an optimised estimate (posterior). As illustrated in Fig. 1A, the posterior falls between the prior and the likelihood and is more precise than either source alone (indicated by its narrower distribution). For instance, when playing against a longstanding training partner, a player may have a strong prior (represented by a narrower prior distribution in Fig. 1A, bottom). In this case, the prior is assigned greater weight, causing the posterior estimate to shift toward it. At the behavioural level, the theory predicts that movements will be systematically biased towards the prior, and the magnitude of this bias should scale with the uncertainty of sensory information (modelled as the width of the likelihood distribution). Over the past two decades, extensive research in the field of motor control (e.g., [45]) and perception (e.g., [46]) has demonstrated that human behaviour aligns with Bayesian integration theory. This principle of reliability-weighted integration is not restricted to the combination of sensory input and prior knowledge; it also applies to the integration of information across different sensory modalities, such as visual–haptic [47] or auditory–visual integration [48].

### Scoping Review of Predictive Processing Studies in Sport

Having provided an overview of the theoretical approach, we now consider the extent to which these concepts have been applied to sport science and summarise the empirical work that has been carried out in this field. We undertook a scoping review of the literature, to explore such applications within sports science research, while also mapping existing knowledge and ‘gaps’ for future studies. From a methodological perspective, scoping reviews provide a valuable exploratory tool for establishing key definitions and conceptual boundaries of a particular topic or field. They are particularly valuable when the existing literature has not been thoroughly reviewed or when the body of work is diverse and complex. Indeed, the primary purpose of a scoping review is to identify the breadth of available evidence—whether quantitative, qualitative, or both—and to visually map or chart these data in a manner that provides a systematic overview of the research landscape. Thus, we conducted a systematic search of extant literature on the PPF in sport science to examine how these cognitive and computational models have been used to understand perceptual-motor skills, decision-making, and physical performance.

## Methods

This review was performed according to the JBI methodology for scoping reviews [49] and the PRISMA Extension for Scoping Reviews (PRISMA-ScR) [50]. A PCC strategy (P-population; C-concept; C-context) was used to define the scope of the review (see pre-registration for more details) and consisted of the following stages: (1) identifying the specific area the review aims to investigate; (2) identifying relevant research papers; (3) organising the data; and (4) compiling, summarising, and reporting the findings. The protocol was pre-registered on the Open Science Framework (OSF). The pre-registration as well as extensive supplementary files can be found online: https://osf.io/mtynv/.

### Identification of the Research Question

The primary aim of the scoping review was to map the current empirical evidence for predictive processing theories in sport science. To this end, our review was guided by the following questions:

1. To what extent have the PPF, or related theories, been applied in sport science?
2. In which domains of sport science have the PPF, or related theories, been applied?
3. What is the extent of evidence for or against PPF hypotheses in sport science?

### Identification of Relevant Studies

An initial search was carried out across the following electronic databases from 1 st of January 1999 until 9th October 2024: EMBASE (via Ovid), Medline (via Ovid), PsycINFO (via Ovid), SPORTDiscus (via EBSCOhost). The search was subsequently updated on 14th November 2025 to capture additional articles in the intervening period. Relevant studies were retrieved using appropriate keywords and Medical Subject Headings (MeSH) terms. We used the following search terms: ((“predictive processing” or “active inference” or “Bayesian Brain” or “predictive coding” or “Bayesian perception” or “prediction error” or “Bayesian decision”)) and (“Sport” or “Athlet*” or “exercise”)). In line with JBI guidelines [49], these search terms were developed through initial searching in Ovid and refinement of terms. The exact terms for each database are detailed in Supplementary File 1.

Studies were considered eligible for inclusion if they satisfied the following inclusion criteria:

1. *Grounding in a theoretical framework.* Studies explicitly referenced predictive processing, active inference, the Bayesian brain, predictive coding, Bayesian perception, Bayesian Decision Theory, or some close variant of these ideas as part of their theoretical framework and as a motivation for the design or hypotheses of the study.
2. *Sport and/or exercise context.* Studies focused on sports, athletic performance, or very closely related domains (e.g., physical strength/endurance, motor control, reaction time, decision-making in sports). Simulated sporting tasks were included (e.g., virtual reality sports). Tasks that were not a sport per se, but used naturalistic sports-related stimuli (e.g., computer-based decision-making with sports videos) or a sub element of a sport (e.g., throwing or catching a ball) were also included.
3. *Empirical research.* Empirical studies (e.g., experiments, interventions, or observational studies) that investigated the PPF in the context of sports or exercise were included. To reflect the broad scope of our scoping review, there were no restrictions imposed on specific study designs, controls, or outcomes. Pilot studies were additionally included.
4. *Peer-reviewed publications.* Only peer-reviewed journal articles (not preprints or conference papers) were considered eligible.
5. *Publication date.* The review was limited to full-text articles published within the last 25 years (since the year 1999) to capture all work since the seminal predictive coding paper by Rao and Ballard [9].
6. *Language.* Only studies published in English were included.

Studies were excluded based on the following exclusion criteria:

1. *Irrelevant theoretical focus.* Studies that did not engage with the PPF or related theories as part of their core framework/rationale (e.g., those that mention them only in passing or only in the discussion).
2. *Non-sport context.* Studies that focused on non-sport settings (e.g., general cognitive tasks, clinically related aptitudes that were not related to physical performance, or simple motor tasks like reaching that are not representative of a sport).
3. *No primary data.* Systematic reviews, meta-analyses, and theoretical papers discussing the PPF in relation to sport science were not eligible, as the scoping review was specifically focused on trial- and study-level evidence. However, the authors retained these designs in the full text stage of article screening as ‘relevant reviews’ during which reviewers completed additional searches of the reference lists.
4. *Non-peer-reviewed publications.* Grey literature, such as theses, dissertations, book chapters, and unpublished studies were excluded.

**Fig. 2:**
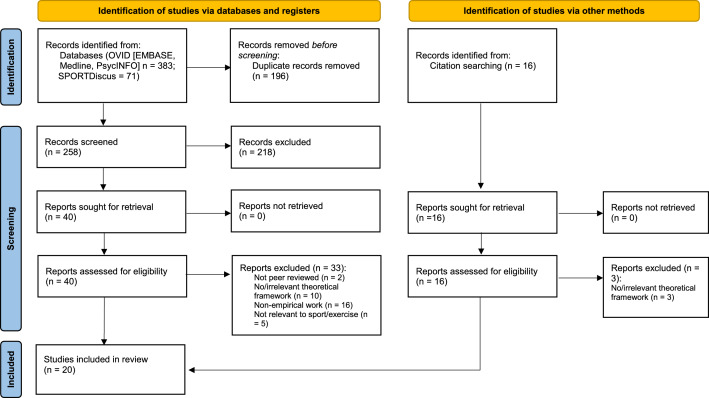
PRISMA flow diagram

Covidence systematic review software (Veritas Health Innovation, Melbourne, Australia) was used for the collation of search results and the screening process. Title and abstract screening were primarily conducted by one reviewer, with 20% screened by a second reviewer to check consistency (89% agreement, Cohen’s *K* = 0.75). The full texts were retrieved for the remaining articles and two reviewers assessed each article in detail against specified inclusion criteria (93% agreement, Cohen’s *K* = 0.85). Study records were screened independently by reviewers for both stages of the selection process, and any disagreements were subsequently resolved through discussion. Paper reference lists and relevant review papers were searched for further relevant articles. This resulted in 20 papers included in the final review. An overview of the search and screening process is shown in the PRISMA flow diagram in Figure 2.

### Collating, Summarising, and Reporting Findings

Data from each of the included studies were independently extracted by members of our review team, using our extraction tool (see Supplementary File 2 on the OSF page for the extraction tool and Supplementary File 3 for the full data extraction spreadsheet: https://osf.io/mtynv/). To understand which areas of sport science the included studies fell into, we classified the studies into at least one subdomain, based on the UK’s Chartered Association of Sport and Exercise Sciences (CASES) divisions: (1) biomechanics and motor behaviour; (2) physical activity for health; (3) physiology and nutrition; (4) psychology; (5) sport and performance. If studies applied to several subdomains, a primary subdomain was denoted by the researcher responsible for data extraction of the study. Free-text summaries were extracted in relation to the main findings of each study. Moreover, we extracted data on: geographical location of the corresponding author; date of publication; participant demographics; the sporting activity used; study design; specific frameworks referred to (e.g., active inference; predictive coding; etc.); context; main outcome measures; hypotheses and their link to the PPF.

## Results

### Study Selection

The initial and updated systematic database searches identified 454 studies. After removal of 196 duplicates, 258 articles were screened for their titles and abstracts. An additional 16 studies were found through citation searching, creating a total of 56 that were evaluated as full texts. Of these, 20 fulfilled the inclusion criteria and were included in the final review (see Supplementary File 4 for a list of articles that were excluded during full-text screening, with reasons provided). The full selection process is illustrated by the PRISMA flow diagram in Fig. 2.

### Study Characteristics

**Fig. 3:**
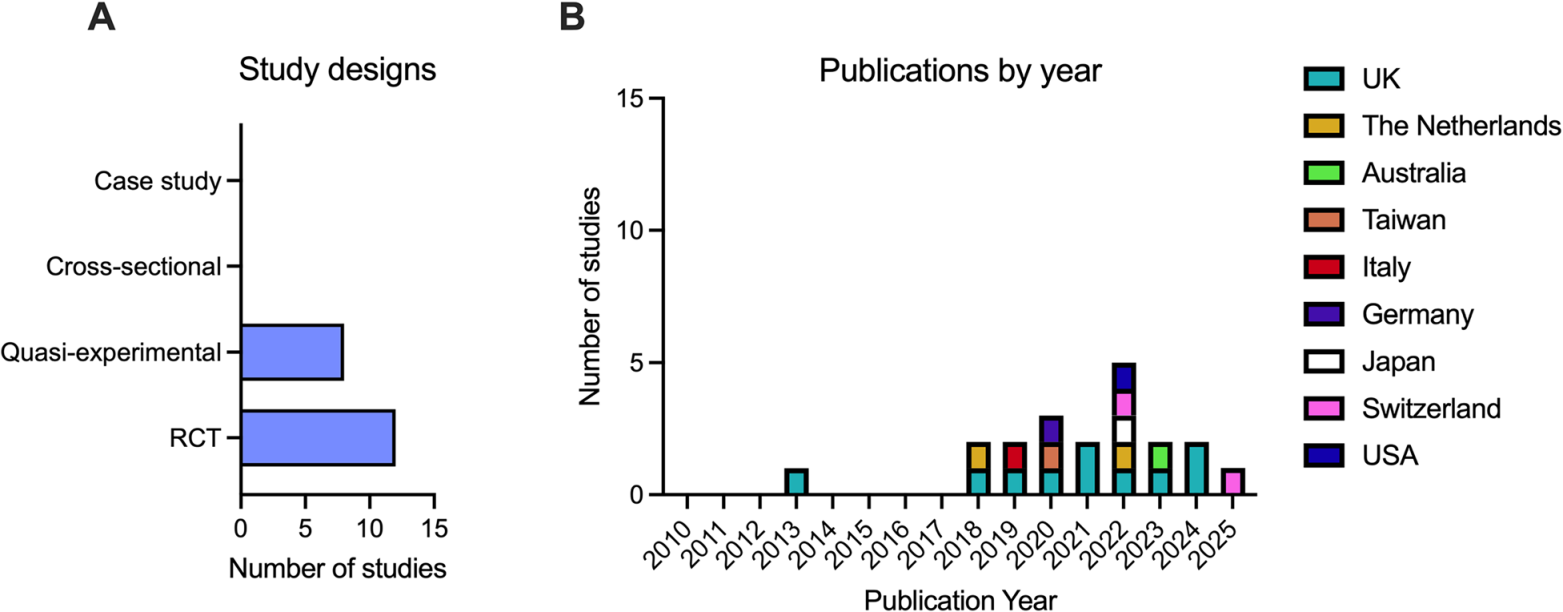
Summary of study characteristics: **A** study design; **B** publications by year. *RCT* randomised controlled trial, *UK* United Kingdom

**Fig. 4:**
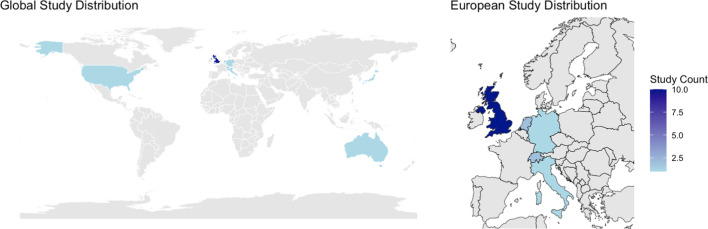
Geographic distribution of studies. Geographic location was coded according to the primary institution of the corresponding author

**Fig. 5:**
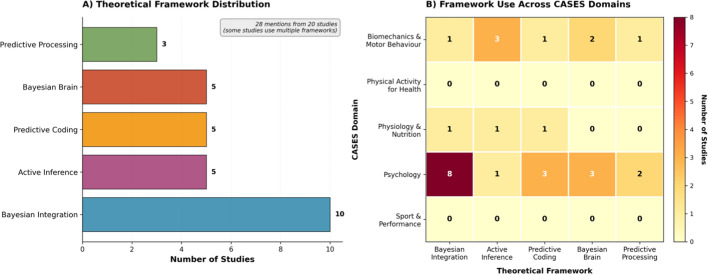
Theoretical framework distribution: **A** shows references to particular frameworks across the included studies; **B** shows how these are distributed across CASES domains

Table 1 provides an overview of the key features of the 20 studies included in the scoping review. The included studies were all published between 2013 and 2025 (see Fig. 3), with all but one (95%) appearing in the last 7 years, highlighting the growing interest in this research area in recent years. All studies in the sample adopted traditional experimental designs. Randomised controlled trials (RCTs) were the most frequently employed methodology, with 12 studies adopting this rigorous approach. Quasi-experimental designs, effectively RCTs but without random assignment, appeared in eight studies. There were no case studies, cross-sectional studies, or longitudinal study designs. Most studies were led by authors from the UK and Europe (see Fig. 4). ‘Bayesian integration’ terminology was the most frequently used in these papers and ‘predictive processing’ was the least (see Fig. 5).

**Table 1:** Summary of studies

Study	BASES domain	Sport/activity	Theoretical* framework	Primary outcome(s)	Main findings	Support for the PPF
Arthur & Harris, 2021 [18]	Biomechanics and Motor Behaviour, Psychology	Squash interception task (in virtual reality)	Bayesian brain; active inference	Fixation height at ball bounce, learning rate	More unpredictable (volatile) sequences of ball trajectories lead to compensatory gaze behaviours and adapted learning rates, consistent with Bayes-optimal behaviours	Supportive
Beck et al. 2025 [70]	Biomechanics and Motor Behaviour, Psychology	Tennis interception (in virtual reality)	Bayesian integration	Predictive gaze location, judgement of bounce location	Participants’ predictive gaze behaviour during interception was systematically biased by prior knowledge. As predicted by Bayesian integration, the reliance on the prior increased with accumulating experience (increasing reliability of the prior) and with higher ball speeds (increasing uncertainty of sensory information)	Supportive
Chen et al. 2020 [59]	Psychology	Baseball video anticipation	Predictive coding	Decision accuracy and reaction time, activation of the action observation network (fMRI)	Consistent with the PPF, a nonlinear (inverted-U) relationship was found between action observation network activity and expertise level	Supportive
Cross et al. 2013 [63]	Psychology	Gymnastics video anticipation	Predictive coding	Prediction accuracy, activation in the action observation network (fMRI)	Training enhanced ability to predict complex action sequences. After training there was increased activation in the action observation network, particularly occipitotemporal and premotor cortices, when participants predicted untrained sequences, indicating that visual experience alone can improve action prediction capabilities	Supportive
Gredin et al. 2018 [54]	Psychology	Soccer defending video anticipation	Bayesian integration	Anticipation efficiency (response accuracy/response time), visual dwell time on the areas of interest	The explicit provision of contextual priors guided the expert's but not novice’s visual attention toward more context-relevant environmental information. The explicit provision of priors enhanced anticipation efficiency on congruent trials among both experts and novices. However, impaired performance on incongruent trials was evidenced only in novices	Supportive
Gredin et al. 2019 [55]	Psychology	Soccer defending video anticipation	Bayesian integration	Response accuracy, anticipation efficiency (response accuracy/response time), verbal reports of thoughts (retrospective)	Contextual priors biased expert soccer players’ anticipation performance and thoughts about positioning in a Bayes-like fashion. However, players became less reliant on contextual priors and unfolding visual information when judgement utility was manipulated	Supportive
Gredin et al. 2020 [56]	Psychology	Soccer defending video anticipation	Bayesian integration	Response accuracy, reaction time, cognitive load (self-report and EEG)	Contextual priors enhanced anticipation performance for congruent trials but there was no effect on performance for incongruent trials. Electroencephalography data suggest that cognitive load may increase when contextual priors are explicitly provided, whereas self-report data suggested a decrease in cognitive load	Supportive
Gredin et al. 2021 [57]	Psychology	Soccer defending video anticipation	Bayesian integration	Response accuracy	Contextual priors biased anticipation judgements towards the prior. This effect was greater when kinematic information was less reliable. When the reliability of kinematic information was high, players used explicit contextual priors of high, but not low, reliability to inform their judgements	Supportive
Harris et al. 2022 [64]	Biomechanics and Motor Behaviour, Psychology	Squash interception task (in virtual reality)	Predictive processing; predictive coding; active inference	Anticipatory eye movements, pupil dilation, model-derived estimates (prior beliefs, volatility, prediction error, and learning rate)	Bayesian learning models better accounted for gaze behaviours than associative learning models. Task-evoked pupil responses tracked prediction errors and learning rates indicating a role in prediction error signalling	Supportive
Harris et al. 2023 [62]	Psychology, Biomechanics and Motor Behaviour	Rugby ball video anticipation	Bayesian brain; active inference; predictive coding	Model estimates of strength of prior beliefs and sensitivity to sensory inputs	Experts were more sensitive to early postural and kinematic cues than novices but there was little difference in their use of priors. Increasing certainty of sensory input shifted experts to rely more on sensory inputs but had little effect on novices	Supportive
Harris et al. 2024 [25]	Biomechanics and Motor Behaviour, Psychology	Squash interception task (in virtual reality)	Active inference	Predictive gaze location, pupil dilation, model-derived estimates (prior beliefs, precision, volatility, prediction error)	Eye movements during interception adhered to environmental probabilities and Bayesian learning models but often reflected a 'best guess' about sensory outcomes instead of minimising distance in a linear sense. Task-evoked pupil dilations signalled prediction errors	Supportive
Helm et al. 2020 [67]	Psychology	Handball anticipation (in virtual reality)	Bayesian integration; Bayesian decision theory	Proportion of throws correctly classified as disguised	Participants used both current sensory and prior contextual information, weighted according to their reliability. Explicitly provided situational probabilities were detrimental to performance when they conflicted with true outcome	Supportive
Heil et al. 2018 [65]	Psychology	Observation of ten-pin bowling videos	Predictive processing	Response reaction time	Participants learned to predict outcomes based on the skill of the observed player and reactions were slowed for unexpected events. Prediction errors relating to low-level features slowed down reporting of higher level features, indicating hierarchical task representations	Supportive
Iodice et al. 2019 [68]	Physiology and Nutrition	Fixed intensity cycling	Predictive coding	Heart rate, perceived effort	Providing non-contingent (faster) cardiac feedback during physical exercise consistently increased the perception of effort compared to a control condition with contingent cardiac feedback. Non-contingent cardiac feedback that was slower than real heart rate did not decrease perceived effort	Supportive
Magnaguagno et al. 2022 [61]	Psychology, Biomechanics and Motor Behaviour	Handball video anticipation	Bayesian integration	Response accuracy	Providing explicit information improved performance when the information was correct and impaired it when incorrect. Over time, the effect of explicit information diminished due to accumulated self-generated knowledge, particularly among experts	Supportive
Mouatt et al. 2023 [66]	Physiology and Nutrition	Fixed intensity cycling in virtual reality	Predictive processing; free energy principle	Power output, heart rate, affective valence, perceived effort, interoceptive accuracy	Individuals with higher interoceptive accuracy perceived higher exertion during illusory downhill cycling versus flat cycling in virtual reality. Meanwhile, those with lower interoceptive accuracy perceived lower exertion in both illusory up and downhill conditions versus flat cycling in virtual reality	Mixed/inconclusive
Nakamoto et al., 2022 [60]	Psychology, Sport and Performance	Baseball speed evaluation in virtual reality	Bayesian integration	Ball-speed judgements (verbal response)	Batters integrated information about ball trajectory in an optimal “Bayesian-like” manner. Exploratory analysis found that more skilled participants integrated the kinematic and ball-speed information more readily than a less skilled group of batters	Supportive
Ridderinkhof et al. 2022 [53]	Psychology	Soccer penalty video anticipation	Predictive processing; Impetus, Motivation, and Prediction in Perception–Action Coordination Theory	Response accuracy, response time	Penalty-kicking practice improved penalty-reading accuracy indicating use of forward models in action anticipation, but motor imagery failed to improve penalty-reading performance. Motor imagery circuits also engaged when saving penalties suggesting forward modelling	Mixed/inconclusive
Smeeton et al. 2024 [52]	Psychology	Tennis video anticipation	Bayesian integration; Bayesian brain	Response accuracy, response time	Priors learned in training resulted in higher accuracy and reduced response time when outcomes were congruent with the prior. Training with both shot direction probabilities and kinematic information lead to greater anticipation skill learning compared to shot outcome probabilities alone	Supportive
Thomas et al. 2022 [58]	Psychology	Soccer defending video anticipation	Bayesian integration	Response accuracy and fixation locations	Skilled players showed superior anticipation but a greater drop in performance immediately following a change in opponent action preferences, suggesting a greater reliance on contextual priors. Skilled participants also fixated more on kinematically relevant areas and on the player off the ball	Supportive

^*^We recorded the terminology that was predominantly used in the paper

*PPF* Predictive Processing Framework, *CASES* Chartered Association of Sport and Exercise Science

### Participant Characteristics

Across the 20 studies, a total of 877 participants were recruited. Of those recruited, 471 were reported as male (54%), 318 reported as female (36%) and 88 recruits did not have their sex reported (10%). No studies reported gender as a separate entity from sex. The mean ± SD age of recruited participants was 20.6 ± 4.9 years. Most studies recruited healthy adults, with many of the participants drawn from university student populations. One study recruited a mixture of adolescent and adult participants [52]. Several studies targeted athletes or individuals with specific sporting expertise. Six studies [53–58] involved athletes participating in soccer, two studies involved baseball players [59, 60], one study involved handball players [61], one study involved rugby players [62], and one involved tennis players [52]. Nine studies either did not explicitly state which activities participants were primarily engaged in or were reported as generally healthy and currently engaged in some form of physical activity with no contraindications to exercise [18, 25, 63–68]. For the seven studies that reported physical activity levels, participants engaged in 8.16 ± 3.52 h/week^−1^ of physical activity. Of the 877 total recruits, a total of 23 full removals of participant data were reported across all 18 studies. Therefore, the final data set comprises 854 participants (a 97% completion/data retention rate). Notably, however, some studies report some data omissions from specific analyses such as psychophysiological measures (e.g., eye-tracking) due to individual trial non-responses or data variability (e.g., [53]). Detailed breakdowns of participant characteristics and study completion rates are provided in Supplementary Files 5 and 6.

### Study Domains

Below we briefly summarise the studies within each of the CASES domains (see also Fig. 5).

#### Biomechanics and Motor Behaviour

Four studies were primarily linked to *Biomechanics and Motor Behaviour* (see Table 1) [18, 25, 64, 69], but also had a secondary classification of *Psychology*, which was a large part of these studies. These four studies [18, 25, 64, 70] all examined whether gaze control in a virtual interceptive task adhered to Bayesian principles in response to manipulation of probabilistic elements of the environment. Beck et al. [70] showed that, when returning tennis serves, participants’ predictive gaze behaviour was systematically biased towards opponents’ preferred serve location (i.e. a learned prior). As predicted by Bayesian integration theory, the reliance on the prior grew with accumulating experience over the experiment (i.e. with increasing reliability of prior information), particularly when ball speeds were high (i.e. with greater visual uncertainty). Harris et al. [64] and Harris et al. [25] found that Bayesian learning models provided good explanations of gaze behaviour and that task evoked pupil dilations responded to probabilistic elements of the task. Finally, Arthur and Harris [18] found that gaze behaviour was also sensitive to the rate of change of probabilities in the environment, indicative of a hierarchical generative model of the task.

#### Physical Activity for Health

No studies in the sample were identified as falling within the *Physical Activity for Health* domain.

#### Physiology and Nutrition

Two studies explored the PPF in the *Physiology* domain, examining whether deceptive sensory information could alter interoceptive inferences. Iodice et al. [68] demonstrated that providing false auditory feedback about heart rate during cycling induced an illusion of increased effort when feedback suggested a faster heart rate, aligning with the PPF. However, when feedback conditions indicated that heart rate was lower than it truly was, they did not observe analogous effects (i.e. lower perceptions of effort), contrary to their initial hypotheses. Mouatt et al. [66] examined whether deceptive visual cues during virtual reality cycling (the appearance of cycling up/downhill) would influence effort and affective valence. The findings showed that the weighting given to visual (exteroceptive) cues, and their subsequent impact on effort and affective valence, depended on the individual’s ability to detect internal body signals.

#### Psychology

Fourteen studies fell primarily into the *Psychology* domain, although several were also linked to *Sport and Performance* and *Biomechanics and Motor Control*, making this by far the most common field of research*.* A large group of these studies examined anticipation performance, and the majority were in ball sports. For instance, five studies by Gredin and colleagues [54–58] examined how athletes integrate contextual priors with kinematic and environmental information to enhance anticipation in sports. Using a video-based decision-making paradigm where participants predicted the actions of opponents in dynamic, sport-specific scenarios, these studies consistently found that expert athletes outperformed novices by effectively combining probabilistic information, situational context, and real-time cues. Participants were found to combine multiple information sources to improve anticipation accuracy and efficiency, consistent with the principles of Bayesian Integration. A further study using a similar video-based occlusion was performed by Smeeton et al. [52], who found evidence that learners update their predictive models in response to probabilistic cues, supporting the role of Bayesian processes in skill acquisition. In handball, using a 360° virtual environment, Helm et al. [67] corroborated earlier anticipation research by demonstrating that participants integrated sensory and contextual information, weighting each based on its reliability. Again in handball, Magnaguagno et al. [61] showed that the performance gain of explicitly proving contextual information (vs. letting players generate it themselves) depends on players’ expertise level, the certainty of provided information and the time to accumulate self-generated knowledge. One study by Nakamoto et al. [60] that focused on the skill of intercepting a moving ball, found that baseball batters in a virtual environment estimated pitch speed by integrating kinematic cues from the pitcher’s motion and ball-flight information, weighting each according to its reliability. Finally, Harris et al. [62] used a Bayesian computational model to estimate the relative weightings of prior and sensory information during video-based anticipation of a rugby ball bounce. It was found that experts were much more sensitive to early postural and kinematic cues than novices but there was little difference in their use of priors. When the certainty of sensory input increased (later temporal occlusion), experts shifted to rely more on sensory inputs but novices did not exhibit these changes.

Four studies examined PPF mechanisms during action observation of sporting tasks. Chen et al. [59] found that action anticipation in baseball batters engages the action observation network (AON) in an expertise-dependent, nonlinear pattern, with intermediate players showing the highest activation, supporting predictive coding frameworks. Ridderinkhof et al. [53] investigated how soccer goalkeepers predict penalty kick directions, finding that practising taking penalty kicks enhances their ability to anticipate opponents' penalties. Functional MRI data revealed that successful predictions were associated with activation in motor imagery circuits. Based on the authors' conclusions, this suggests that simulating the observed action may facilitate understanding and anticipation of others’ intentions, thereby evidencing the role of forward models in action anticipation. Cross et al. [63] showed that visual training enhances activation in the AON during the prediction of complex, occluded movements (gymnastics), with stronger responses for less familiar sequences, which was again taken as support for predictive coding models. Finally, Heil et al. [65] investigated how hierarchical predictive processing influences the perception of actions performed by agents, demonstrating that observers’ expectations about an agent’s expertise affect their predictions of action outcomes. Additionally, it was observed that prediction errors relating to action outcomes slowed participants' responses about the agent’s identity (as a novice or expert), indicating hierarchical passing of prediction errors slowing responses at higher levels of representation.

#### Sport and Performance

None of the studies received a primary classification of *Sport and Performance*, but several studies investigating anticipation and interceptive performance (see Sect. "Psychology") could also be linked to this domain.

### Evidence for Predictive Processing Theories

Of the 20 studies, 18 (90%) provided evidence clearly in support of PPF hypotheses (see Table 1). The remaining two studies returned mixed or inconclusive findings, although were still broadly supportive of PPF hypotheses. Firstly, Mouatt et al. [66] found that illusory downhill cycling reduced affective valence, consistent with the PPF, but illusory uphill did not improve it. Secondly, Ridderinkhof et al. [53] tested whether penalty *taking* and motor imagery would improve penalty *saving* due to forward modelling of the movements, but only found support for benefits of penalty taking, not motor imagery. Overall, the body of evidence reviewed strongly supports the applicability of the PPF to sport science domains.

## Discussion

This scoping review explored the existing literature on the PPF applied to sport and exercise domains. We sought to understand where and how ideas derived from the PPF have been applied and tested, and the extent to which studies in a sporting context have generated additional evidence for or against the approach. The discussion is hereafter organised around our three research questions. We then provide an overview of some relevant review papers which were identified in the searches but did not meet inclusion criteria. Finally, we explore how future research in the sport and exercise domain can rigorously address questions grounded in the PPF, advancing the empirical testing of these theories.

### To What Extent Have Predictive Processing, or Related Theories, Been Applied in Sport Science?

Our results show that the application of the PPF and related theories within sport science has gained notable momentum in recent years but remains limited. The 20 studies identified in this review span various subfields, including anticipation, interception of moving targets, and action observation, with most focusing on cognitive and perceptual processes underpinning skilled performance. While these studies demonstrate an emerging interest in using the PPF to study phenomena such as the integration of contextual priors, sensory weighting, and hierarchical passing of prediction errors, their distribution across topics is uneven, focusing on a relatively narrow range of themes. Furthermore, the use of the PPF remains largely confined to experimental designs investigating short-term mechanisms, with no longitudinal or applied studies exploring the broader implications of the PPF for skill acquisition or training interventions. Therefore, despite growing interest, the application of PPF in sport science is still in its developmental stages.

### In Which Domains of Sport Science Have Predictive Processing, or Related Theories, Been Applied?

We found PPF ideas have been primarily applied within the domains of Psychology and Biomechanics in sport science, with a particular focus on anticipation, interception, and action observation. Although assigning each study a single primary subdomain inevitably simplifies cases of genuinely multidisciplinary work, this pragmatic step allowed us to chart the landscape of research activity, while still acknowledging that many studies legitimately spanned multiple subdomains. Of the 20 studies included in this review, the vast majority (18) were classified as Psychology, often overlapping with Biomechanics and Motor Control, while only two studies addressed Physiology and none explicitly targeted Physical Activity for Health or Sport and Performance as primary domains. Although this imbalance is perhaps unsurprising, given the neuroscientific origins of the PPF, it sits in contrast with the broad scope and ‘applicability’ of these conceptual approaches. The free energy principle and active inference [51] present plausible and unified explanations for extensive physiological systems, functions (e.g., cardiorespiratory control), and patterns of human behaviour (e.g., action responses, motor planning and execution, and the pursuit of short- vs long-term goals; see [27] and [71] for further discussions). Indeed, homeostatic and allostatic control are the ultimate goals of prediction error minimisation in these theories. The relative absence of studies in biomechanics and physiology therefore reflects a research gap rather than lack of theoretical relevance.

As discussed, the most prominent application of the PPF has been in the area of anticipation [52, 54–58, 61, 62, 67]. These studies effectively illustrate the concepts of Bayesian integration and precision weighting in sports-related tasks, whereby the assimilation of information for perceptual judgements or decision-making is conducted according to the (perceived) precision of those sources [41, 42, 45] (see Fig. 1A). Several studies on interception also support Bayesian integration processes in learning situational probabilities and making probability-based decisions and/or judgements [25, 60]. These studies have also indicated that performers will adjust their gaze behaviours in line with such learned probabilities [18, 25, 64, 70], in support of key active inference hypotheses (i.e. that actions are used to minimise prediction errors) [10]. In this way, the field of sport science is uniquely suited to investigate how complex and naturalistic human actions conform to ‘free energy’ principles and could therefore be central to future developments of the PPF.

From a more exercise-focused perspective, Iodice et al. [68] and Mouatt et al. [66] examined the idea that interoceptive experiences, such as effort and affective valence, arise from Bayesian inference processes (see [33]), where the brain generates and updates predictions about internal bodily states by integrating prior expectations with sensory feedback. Notably, these studies found that effort and affective valence were susceptible to manipulation through the provision of deceptive sensory cues. Although such findings largely echo those already reported in endurance-focused research (e.g., [72, 73]), the novel application of Bayesian inference processes, which can be prospectively and individually modelled, is highly significant in this context. Manipulating interoceptive cues in line with individualised, neuro-computational models could, therefore, be a valuable tool in sport science, offering new strategies to enhance performance, optimise training, and improve athletes’ resilience to physical and mental fatigue. For instance, providing athletes with subtly biased feedback about their heart rate or breathing patterns during training could shift their interoceptive predictions, potentially allowing them to sustain higher effort levels or manage perceived exertion more effectively.

### What is the Extent of Evidence for or Against Predictive Processing Hypotheses in Sport Science?

The application of the PPF in sport science is supported by a relatively small but growing body of research. Eighteen of the 20 studies [18, 25, 52, 54–65, 67, 68, 70] provided evidence that supported the PPF, either through testing of highly specific hypotheses relating to hierarchical predictions or active inference processes in perception and action, or more broadly showing anticipatory behaviour and the use of prior knowledge. These studies reveal that in many cases researchers are developing and testing specific and falsifiable hypotheses to advance this theoretical area. Gaps remain, however, in comparing predictive-processing-specific mechanisms to alternative theories, and in testing these frameworks across more diverse sport-specific contexts.

One concern with the papers included in the review is the absence of any negative findings providing evidence *against* the PPF. Just two studies [53, 66] reported failing to support all of their hypotheses, but even these papers still found some support for the PPF. On the one hand, this lack of contradictory findings could indicate some level of publication bias. Alternatively, it could indicate limitations in the scope or methodology of the reviewed studies. For instance, studies may have predominantly focused on conditions or contexts where the PPF is expected to succeed, rather than testing new hypotheses or challenging boundary conditions. An example of this, is how many of the studies in the review addressed the integration of contextual priors with current sensory observations during anticipation. A strong body of evidence already exists outside of sport science indicating that Bayesian integration is a compelling explanation of how we combine information sources in situations of uncertainty [41, 45, 74]. These studies, therefore, provide a useful extension of these findings to sport, but could be considered more ‘conservative’ in terms of the hypotheses they are testing.

A recent review of broader, non-sporting evidence [75] concluded that while a variety of findings support predictive coding mechanisms and active inference models of behaviour, some of these can also be explained by alternative theories and that more direct comparisons to other competing accounts are required. A similar point can be made about some studies in the present review. For instance, studies of anticipation [52, 54, 55] have demonstrated an influence of prior expectations on performance and a greater weighting applied to more reliable information, but these findings can also be explained with other mechanisms (e.g., simple rules or heuristics relying on the most salient or recent information, regardless of its probabilistic value). Given that very few of the studies in the review directly compared the PPF to other plausible accounts, the degree of support for these approaches in sport therefore remains somewhat unclear, at least from an explanatory, mechanistic perspective.

Some studies did, however, provide more compelling evidence for PPF mechanisms, by testing hypotheses relating to very specific or unexpected phenomena (e.g., [25, 53, 59, 64, 65]). For instance, Harris et al. [64] used computational modelling approaches to determine whether Bayesian learning models provided a better account of the trial-to-trial changes in individual eye movements than traditional reinforcement learning models. This computational approach provides evidence that directly examines competing theories and PPF-specific constructs (e.g., volatility beliefs) in an attempt to adjudicate between pertinent theoretical explanations.

In addition to identifying and testing more specific, competing hypotheses, it is also valuable for our field of sport science to recognise convergences between distinct theoretical frameworks. For instance, Bayesian integration, as a pertinent PPF concept, is also central to optimal feedback control theory [76], which is a prominent framework in motor control [44, 77, 78]. While differences between optimal feedback control and active inference exist [79], they share the theoretical core of viewing perception and action as fundamentally probabilistic. Specifically, based on both frameworks, the hypothesis can be derived that humans integrate information sources in a Bayesian (reliability-based) fashion. Thus, rather than choosing a theoretical ‘camp’ (e.g., optimal control vs. active inference), we argue that it is more productive for our field to test specific hypotheses (e.g., relying on the most recent information [heuristic rule] vs. on probabilistic integration [Bayes’ rule]). Overall, convergences at the level of key concepts are, in our view, good news for joint and coherent progress in understanding sport-relevant phenomena.

A recent review by Beck et al. [23], which included studies outside of the scope of the current review, concluded that there was growing evidence for precision weighting mechanisms at play in sporting tasks, both relating to Bayesian integration of prior knowledge and multisensory integration. They also found some (but less conclusive) evidence for related uncertainty-based scaling mechanisms, such as impedance control to handle unexpected uncertainty [80] and risk optimisation to deal with uncertainty in motor outcomes due to inherent motor noise [69]. Beck et al. [23] propose that studies that derive specific and testable hypotheses—ideally, through explicit computational models—may be most beneficial in future for understanding uncertainty-related effects. In relation to our wider assessment of the PPF in sport, we come to similar conclusions. Taken together, while there is a growing body of work that is consistent with PPF ideas, more specific testing of predictions derived directly from theory, across a wider range of phenomena, is needed.

A broader issue underlying these patterns of evidence is the status of the PPF itself. Predictive processing is best understood as an overarching *framework*, that is a set of general principles about hierarchical prediction and error minimisation, rather than a fully specified scientific theory. As such, it does not generate precise behavioural predictions on its own, but rather provides a conceptual scaffold from which researchers must derive domain-specific, falsifiable hypotheses. This requirement helps explain why many existing studies in sport have tested relatively general ideas (e.g., Bayesian integration or reliability weighting) rather than more discriminative predictions. The future progress of PPF in sport science (and beyond) will therefore depend on refining these broad principles into specific causal theories that can be adjudicated against plausible alternatives. In this sense, the empirical success of the PPF will rest not on any single result, but on whether these more precise derivations consistently succeed, or fail, across a range of sporting phenomena.

### Summary of Relevant Review Papers

The database searches uncovered several review papers that have applied the PPF to sport, but did not feature any empirical data. These papers therefore did not meet the inclusion criteria but provided critical background in writing this review. We have therefore provided a full list and a narrative summary of these reviews in Supplementary File 7 (available from the OSF page: https://osf.io/mtynv/). Briefly, several of these review papers have applied predictive processing and active inference frameworks to sport, offering insights into phenomena such as choking [22, 81], flow states [82], fatigue [15], and motor imagery [21]. For instance, Harris et al. [22] and Cappuccio et al. [81] link choking under pressure to disruptions in sensorimotor predictions that force athletes to rely on maladaptive sensory feedback, while reviews on flow [82] suggest that high precision in second-order beliefs underpins the smooth execution characteristic of athletes “in the zone”. Other work, such as that by Greenhouse-Tucknott et al. [15], conceptualises fatigue as the brain’s response to persistent interoceptive prediction errors. Ridderinkhof also proposes *IMPPACT* (Impetus, Motivation, and Prediction in Perception–Action Coordination Theory) [83], which extends predictive-processing principles to broader perception–action coordination. Finally, recent efforts have even begun exploring how the PPF can inform coaching practices [84]. Collectively, these reviews highlight the promise of the PPF as a unifying model in sport performance research and invite further empirical investigation.

### Questions to be Answered for the PPF

To help steer future investigations, we outline several empirical predictions that can be tested in line with the core tenets of the PPF. Rigorous testing of these hypotheses is essential for advancing our mechanistic understanding of the PPF in sport. By scrutinising these central concepts, we can apply these ideas to the diverse domains of sport science and perhaps evolve the framework in this context:

1. *Predictions and Prediction Errors.* The brain continuously generates predictions about sensory input and updates its internal models in response to prediction errors—discrepancies between expected and actual sensory feedback [85]. Future research can investigate the role of learned expectations in shaping perception and action, as well as how experimentally induced prediction errors drive learning and adaptation. In anticipation studies, this could involve examining whether athletes with greater domain expertise show reduced neural prediction errors when observing familiar game situations, or whether manipulating the reliability of kinematic cues systematically affects prediction error magnitude and subsequent motor corrections. Similarly, studies of effort perception could test whether experimentally induced mismatches between expected and actual physiological feedback (e.g., false heart rate information) generate prediction errors that recalibrate effort judgements.
2. *Hierarchical Generative Models.* The brain operates using hierarchical generative models that predict sensory input at multiple levels of abstraction [42]. Higher levels generate broad, abstract predictions, while lower levels focus on more specific sensory details. This hierarchy can be empirically tested by examining how perturbations at one level affect predictions and errors at other levels. For example, higher level beliefs that the environment is highly changeable (volatile) should affect lower level sensorimotor behaviours that should account for additional uncertainty [86]. In anticipation tasks, this hierarchical organisation predicts that high-level contextual information (e.g., score, game situation, opponent tendencies) should modulate the weighting of low-level kinematic cues; hence, athletes might rely more heavily on early kinematic information in high-pressure contexts where prediction errors are costly. Similarly, in pain and effort perception, this predicts that higher-level beliefs about the training context (e.g., ‘this is normal training soreness’ vs. ‘this might be an injury’) should influence how interoceptive signals at lower levels are interpreted and integrated, potentially explaining placebo and nocebo effects in sport.
3. *Encoding Probabilities.* The brain encodes probabilistic information about internal and external states, representing not just what it expects, but how strongly (e.g., reliability of effort signals, or certainty about injury-related pain versus muscle soreness). This tenet can be empirically tested by examining how shifts in probabilistic representations influence predictive accuracy and behaviour. For instance, studies could explore how the brain updates predictions when the reliability of sensory inputs changes or when contextual cues with varying uncertainty levels are introduced. In the sport anticipation literature, this could explain why athletes show different response patterns to high-probability versus low-probability action sequences, and suggests that manipulating the statistical structure of practice environments should systematically affect both anticipatory performance and confidence judgements.
4. *Free Energy and Prediction Error Minimisation. *Organisms strive to minimise free energy, reducing both prediction errors and uncertainty about internal states. This principle governs perception, action, and cognition, suggesting that behaviour and neural responses should align with free energy minimisation (e.g., optimising movement efficiency, regulating effort expenditure, managing pain and injury risk). Empirical tests could analyse how individuals adjust their actions and neural activity across tasks to optimise prediction accuracy. For example, studies could examine whether action selection in sports, such as penalty kick direction, follows computational models of optimal probabilistic decision-making. This framework predicts that athletes should exhibit behavioural and physiological patterns consistent with minimising surprise—for example, by adopting movement strategies that maintain proprioceptive predictions within expected ranges, or by modulating arousal/attention to increase precision of task-relevant sensory channels during critical performance moments. In the effort and fatigue domain, this notion predicts that athletes might prematurely terminate exercise when the discrepancy between predicted and actual interoceptive states becomes excessively costly to resolve.
5. *Precision Weighting.* The brain assigns different levels of precision to predictions and sensory inputs according to their perceived reliability [42]. This determines how much weight is given to different types of information in updating models. Changes in the precision of predictions and sensory inputs should therefore affect how errors are processed and incorporated into internal models. Experimental research can investigate how manipulating the reliability of sensory inputs or expectations affects learning, adaptation, and decision-making in cognitive and perceptual tasks (e.g., [45, 87]).

## Conclusions

In conclusion, this scoping review highlights both the promise and current limitations of applying the PPF to sport and exercise science. While the body of evidence supporting key principles of predictive processing is growing, much of it remains preliminary and confined to isolated domains. Supportive findings have begun to emerge, but gaps persist in testing predictive-processing-specific predictions against competing theories and extending research into more applied and longitudinal contexts. As this field matures, there is a pressing need for rigorous, theory-driven empirical studies that test the fundamental tenets of the PPF across a broader range of sporting phenomena. By addressing these challenges, future research can unlock the full potential of predictive processing ideas to transform our understanding of human performance in sport.

## Data Availability

There are no new data associated with this article, but summary data sheets and code are available from: https://osf.io/mtynv/.

## Abbreviations

AON: Action observation network
CASES: Chartered Association of Sport and Exercise Sciences
IMPPACT: Impetus, motivation, and prediction in perception–action coordination theory
MeSH: Medical subject headings
OSF: Open Science Framework
PCC: Population, concept, context
PPF: Predictive processing framework
PRISMA-ScR: Preferred Reporting Items for Systematic Reviews and Meta-Analyses Extension for Scoping Reviews
RCT: Randomised controlled trial
SD: Standard deviation
